# Single-Walled Carbon Nanotubes Modify Leaf Micromorphology, Chloroplast Ultrastructure and Photosynthetic Activity of Pea Plants

**DOI:** 10.3390/ijms22094878

**Published:** 2021-05-05

**Authors:** Violeta Velikova, Nia Petrova, László Kovács, Asya Petrova, Dimitrina Koleva, Tsonko Tsonev, Stefka Taneva, Petar Petrov, Sashka Krumova

**Affiliations:** 1 Institute of Plant Physiology and Genetics, Bulgarian Academy of Sciences, Acad Georgi Bonchev Str. Bl. 21, 1113 Sofia, Bulgaria; petrova_assya@abv.bg; 2 Institute of Biophysics and Biomedical Engineering, Bulgarian Academy of Sciences, Acad Georgi Bonchev Str. Bl. 21, 1113 Sofia, Bulgaria; zlatkova.nia@gmail.com (N.P.); tsonev@gmail.com (T.T.); sgtaneva@gmail.com (S.T.); 3 Biological Research Center, Institute of Plant Biology, Temesvári krt. 62, 6726 Szeged, Hungary; kovacs.laszlo@brc.hu; 4 Faculty of Biology, Sofia University “St. Kliment Ohridski”, 8 Dragan Tsankov, 1164 Sofia, Bulgaria; koleva@biofac.uni-sofia.bg; 5 Institute of Polymers, Bulgarian Academy of Sciences, Acad Georgi Bonchev Str. Bl. 103, 1113 Sofia, Bulgaria; ppetrov@polymer.bas.bg

**Keywords:** carbon nanotubes, thylakoid membranes, photosynthesis, photosystem II, leaf pigments, waxes

## Abstract

Single-walled carbon nanotubes (SWCNTs) emerge as promising novel carbon-based nanoparticles for use in biomedicine, pharmacology and precision agriculture. They were shown to penetrate cell walls and membranes and to physically interact and exchange electrons with photosynthetic complexes in vitro. Here, for the first time, we studied the concentration-dependent effect of foliar application of copolymer-grafted SWCNTs on the structural and functional characteristics of intact pea plants. The lowest used concentration of 10 mg L^−1^ did not cause any harmful effects on the studied leaf characteristics, while abundant epicuticular wax generation on both leaf surfaces was observed after 300 mg L^−1^ treatment. Swelling of both the granal and the stromal regions of thylakoid membranes was detected after application of 100 mg L^−1^ and was most pronounced after 300 mg L^−1^. Higher SWCNT doses lead to impaired photosynthesis in terms of lower proton motive force generation, slower generation of non-photochemical quenching and reduced zeaxanthin content; however, the photosystem II function was largely preserved. Our results clearly indicate that SWCNTs affect the photosynthetic apparatus in a concentration-dependent manner. Low doses (10 mg L^−1^) of SWCNTs appear to be a safe suitable object for future development of nanocarriers for substances that are beneficial for plant growth.

## 1. Introduction

Carbon nanotubes (CNTs) exhibit unique physical properties (thermal and electrical conductivity, elasticity and resilience) that can offer multiple possibilities for innovations in everyday life, science and technology [1,2]. In the field of plant biology and precision agriculture, CNTs are already used as smart targeted delivery systems of biomolecules, agrochemicals or for plant bioengineering [3,4,5], as well as to detect and prevent plant diseases [6], and consequently to increase agriculture output [7]. Recent studies have demonstrated that CNTs could also be successfully used as nanobionic sensors to study how plants respond to stress [8]. CNTs have been studied in plant science from different aspects: as a growth promoter, as carrier of genetic material, as potential agents with cellular and genetic toxicity due to the induced oxidative stress, as well as hazard agents in ecosystems [9,10].

The potential application of CNTs is based on their capability to penetrate through cell walls and plant cell membranes. Liu et al. demonstrated for the first time the ability of single-walled carbon nanotubes (SWCNTs) to penetrate cell walls and cell membranes of intact *Nicotiana tabacum* plant cells and proposed that SWCNTs could deliver different cargoes into different plant cell organelles [11]. The authors suggested that SWCNTs can penetrate both the hard cell wall and the cell membrane most probably mediated by means of fluid-phase endocytosis. Later, it was demonstrated that SWCNTs can be absorbed by roots and cells of a number of crop species (barley, corn, rice, soybean, switchgrass, tomato) as well as of plant cell cultures (tobacco callus) [7]. Lew et al. [5] experimentally validated the model of lipid exchange envelope penetration mechanism for protoplasts and found that the distribution of DNA-wrapped SWCNTs within plant protoplasts was controlled mainly by the size and the magnitude of the ζ-potential of nanoparticles. This study also revealed that glycerolipids (the predominant lipids in chloroplast membranes) exhibit a stronger nanoparticle–lipid interaction than phospholipids (the major constituent of protoplast membranes). Giraldo et al. [12] demonstrated that SWCNTs can passively penetrate through the chloroplast membrane via diffusion and are able to influence photosynthetic activity by supplying electrons into the photosynthetic electron transport chain. Chloroplasts injected with SWCNTs were found to photosynthesize more efficiently than non-infiltrated chloroplasts [12]. Han et al. [13] demonstrated the possibility to design SWCNTs that can absorb in a desired light window while concentrating excitons. Thus, this property of SWCNTs allows them to absorb and convert solar energy into excitons and to efficiently transfer electrons to the photosynthetic apparatus. A molecular dynamics study showed that SWCNTs may interact with photosynthetic pigments (chlorophylls and xanthophylls) and this interaction was stronger on the inner surface of SWCNTs than on the outer surface [14]. It was also demonstrated that chlorophylls interact via their phytol chain, while xanthophylls form π–π stacking and semi-hydrogen bonds. Several studies have shown that photosynthetic reaction centers can be attached to CNTs and exchange electrons with those types of materials [15,16,17,18]. Data presented in [15] not only provide direct evidence that the photosynthetic reaction center proteins can be attached to SWCNTs, but also support the idea that SWCNTs could stabilize light-induced charges while converting photosynthetic energy into a usable chemical form. In the context of artificial photosynthesis, the special electronic properties of the SWCNT/reaction center complex are very promising for energy conversion and storage. Indeed, [19] constructed a hybrid assembly of SWCNTs coupled with light-harvesting photosynthetic proteins and demonstrated that such materials can be highly efficient energy acceptors.

To the best of our knowledge, studies addressing the possible interactions between carbon-based nanomaterials and photosynthetic machinery have so far only been performed in isolated cells or chloroplasts. Therefore, this work is the first to show experiments carried out with intact plants sprayed with SWCNTs. This type of SWCNT application was chosen in order to assess the effect of SWCNTs on intact plant systems, where all metabolic processes actively function. It was specifically aimed at answering the question if foliar application of SWCNTs provokes changes on structural and functional levels of organs (leaves) and photosynthetic organelles (chloroplasts). We hypothesized that (1) the application of SWCNTs would result in structural and functional changes of the photosynthetic apparatus and that (2) the effects of SWCNTs might be dose-dependent allowing determination of concentrations suitable for targeted delivery and plant priming or that they could exert toxic effects. Determining the range of optimal concentrations would be essential for the future development of SWCNT-based nanocarriers as delivery systems that can also moderate plant growth and development and would result in important consequences for plant nanotechnology. The priming dose could help plants to adapt to different environmental conditions (as observed for a variety of mild stresses).

## 2. Results

### 2.1. SWCNTs Affect Leaf Morphology and Chloroplast Ultrastructure

**Figure 1:**
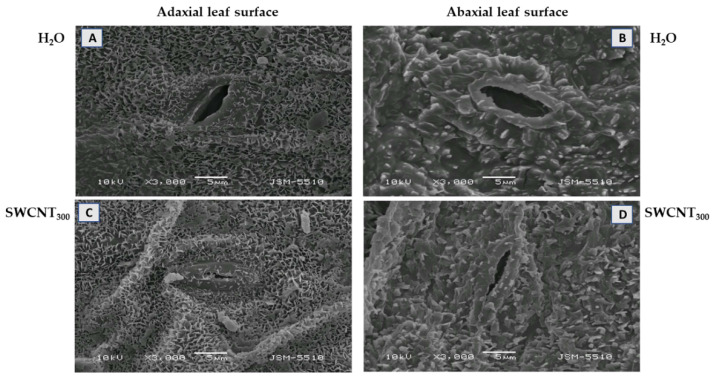
Effect of SWCNTs on leaf micromorphology. Cuticle and epicuticular waxes at the adaxial (**A**,**C**) and abaxial (**B**,**D**) leaf surface of pea plants seven days after spraying with H_2_O (**A**,**B**) and SWCNT_300_ (**C**,**D**).

The changes in leaf morphology in pea plants after spraying with 10, 100 and 300 mg L^−1^ SWCNTs (denoted hereafter as SWCNT_10_, SWCNT_100_ and SWCNT_300_, respectively) and the corresponding concentrations of biocompatible copolymer “Pluronic” P-85 (P_10_, P_100_ and P_300_, respectively) are shown in Figure 1. Morphological analysis of pea leaf surfaces and visualization of leaf cuticular and epicuticular waxes were performed by scanning electron microscopy (SEM). Pea leaf surfaces had a smooth cuticle with epicuticular crystalloid waxes. On both epidermises in control plants, the outer elastic edge of stomata had a typical elongated elliptical shape and the amount of epicuticular waxes was minimal (Figure 1A,B). On the adaxial cuticle, the epicuticular waxes were deposited in the form of tiles of different sizes and orientations, nearly uniformly across the entire leaf surface (Figure 1A), while the abaxial cuticle was dominated by coarse-grained waxes (Figure 1B).

Substantial changes in leaf morphology of both epidermises were observed after spraying of the plants with SWCNT_300_, i.e., the outer elastic edge was more strongly cutinized and stomata were more closed (Figure 1C,D). Furthermore, the epicuticular waxes were of the same type as in the control, but in a larger amount, including on the elastic edges. No significant changes in leaf morphology were identified after copolymer (P_10_, P_100_ and P_300_) (data shown for P_300_, Figure S1) and SWCNT_10,100_ treatments (data not shown).

**Figure 2:**
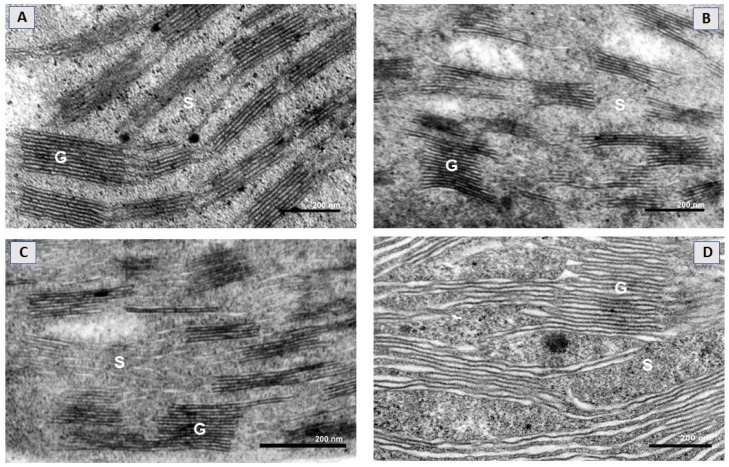
Representative electron micrographs of a chloroplast cross-section taken from intact pea leaves seven days after spraying with H_2_O (**A**) and different concentrations of SWCNTs (**B**–**D**). Chloroplast micrographs illustrate organization of the thylakoid membrane system in the control (**A**) and in SWCNT_10_- (**B**), SWCNT_100_- (**C**) and SWCNT_300_- (**D**) treated plants. G—grana, S—stroma lamellae.

The mesophyll chloroplasts of control plants were characterized by a well-developed inner thylakoid membrane system that consisted of relatively short (~4–6 stacked thylakoid regions) and medium-high (up to 15 of them) grana connected with stroma lamellae of different length (Figure 2A). The chloroplast thylakoid membrane system was affected by SWCNT treatments in a concentration-dependent manner. In SWCNT_10_ plants, chloroplast ultrastructure remained unchanged compared to the controls (Figure 2B), while in SWCNT_100_ samples, granal thylakoids and stroma lamellae were swollen along their entire length, but no apparent fragmentation or any other forms of destruction were found (Figure 2C). In SWCNT_300_ plants, the chloroplast thylakoid membrane system was the most affected and the swelling of both stroma lamellae and granal thylakoids was more pronounced than at lower concentrations.

The stroma lamellae/grana interface in SWCNT_300_ was not clearly defined and apparently unstacked membrane regions coexisted with granum-like regions with a partially preserved stacked configuration (Figure 2D). At this concentration, single chloroplasts with a destroyed envelope were also occasionally found (data not shown). No substantial changes in the chloroplast ultrastructure of P-treated plants were found as compared to control plants (Figure S2).

### 2.2. SWCNTs Affect CO_2_ Assimilation, Photosynthetic Light Reactions and Membrane Proton Permeability

**Figure 3:**
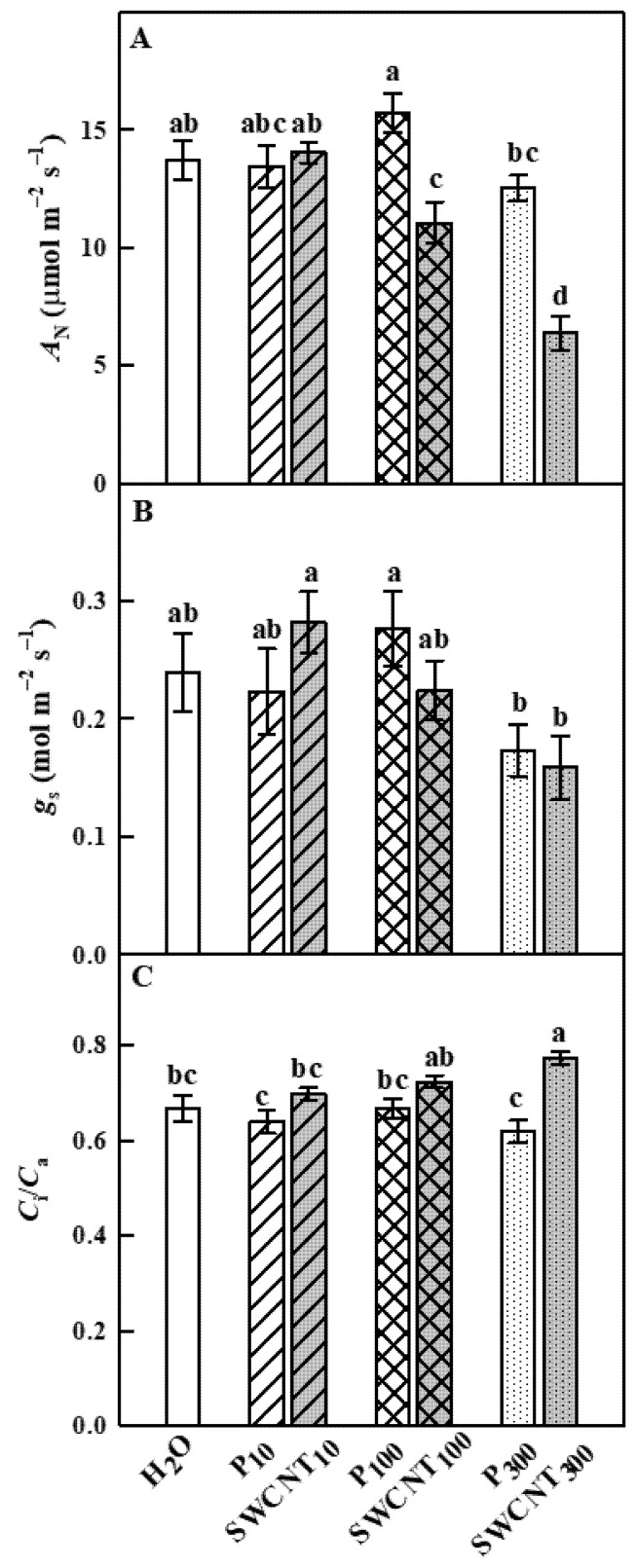
Effect of SWCNTs on photosynthetic gas exchange. Net photosynthesis rate (*A*_N_, **A**), stomatal conductance (*g*_s_, **B**) and intercellular CO_2_/ambient CO_2_ concentration ratio (*C*_i_/*C*_a_, **C**) of pea plants seven days after spraying with “Pluronic” P-85 (P_10,100,300_) and SWCNT_10,100,300_. Plants sprayed with H_2_O served as the control. Data are means ± SE (*n* = 6). One-way analysis of variance (ANOVA) with a between-subjects factor was performed to assess statistically significant mean differences. Mean separation was performed using a Tukey’s test and statistically different means at the 5% level (*p* < 0.05) are indicated with different letters.

The changes in photosynthetic gas exchange parameters in pea leaves after spraying with different concentrations of SWCNTs and biocompatible copolymer “Pluronic” P-85 are shown in Figure 3.

No substantial differences between control (H_2_O-treated), P- (at any concentration) and SWCNT_10_-treated plants were observed in net photosynthesis rate (*A*_N_), stomatal conductance (*g*_s_) and intercellular CO_2_/ambient CO_2_ concentration ratio (*C*_i_/*C*_a_) parameters (Figure 3). *A*_N_ values of SWCNT_100_- and SWCNT_300_-treated plants were significantly lower compared to the control plants and the corresponding P concentrations (Figure 3A). Stomatal conductance (*g*_s_) was not significantly different in the various SWCNT- and P-treated plants compared to the controls, as well as between SWCNT and P treatments for each of the applied concentrations, although it exhibited lower mean values for SWCNT_300_ and P_300_ variants (Figure 3B). The C_i_/C_a_ ratio was significantly higher than the control only for the SWCNT_300_ treatment (Figure 3C). These results strongly suggest that a part of *A*_N_ reduction in SWCNT_300_-treated plants was not related to stomatal limitations, which is in line with the strongest inhibition of chlorophyll (Chl) fluorescence parameters measured in SWCNT_300_ plants (see below).

**Figure 4:**
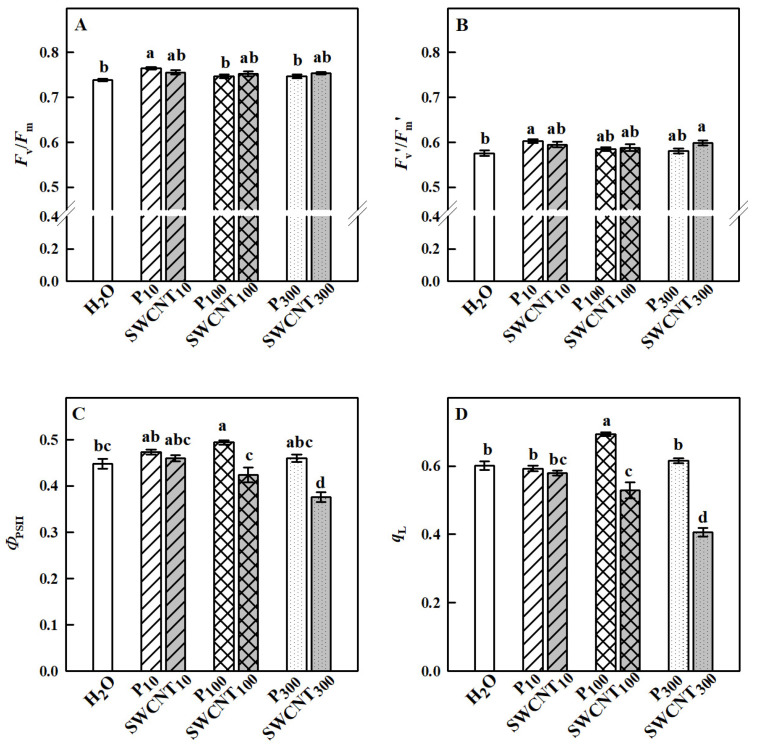
Effect of SWCNTs on chlorophyll fluorescence parameters. Maximum efficiency of PSII photochemistry in dark- (*F*_v_/*F*_m_, **A**) and light-adapted (*F*_v_′/*F*_m_′, **B**) leaves, actual photochemical efficiency of PSII (*Φ*_PSII_, **C**) and fraction of open PSII centers (*q*_L_, **D**) of pea plants seven days after spraying with different concentrations of “Pluronic” P-85 (P) and SWCNTs. Plants sprayed with H_2_O served as the control. Data are means ± SE (*n* = 6). One-way analysis of variance (ANOVA) with a between-subjects factor was performed to assess statistically significant mean differences. Mean separation was performed using a Tukey’s test and statistically different means at the 5% level (*p* < 0.05) are indicated with different letters.

The maximum efficiency of PSII in dark-adapted plants (*F*_v_/*F*_m_) remained unaffected in all SWCNT- or P-85-treated plants compared to the controls with the only exception of P_10_ plants, where the highest values of *F*_v_/*F*_m_ were registered (Figure 4A). The maximum PSII efficiency in light-adapted leaves (*F*_v_′/*F*_m_′) which provides an estimate of the maximum efficiency of PSII photochemistry, i.e., the PSII operating efficiency when the maximum number of PSII centers is open [20], was significantly higher in P_10_ and SWCNT_300_ compared to the controls, while the other treatments did not cause any substantial effect (Figure 4B).

*F*_v_′/*F*_m_′ values did not differ between the different SWCNT and P-85 treatments with corresponding concentrations. The actual quantum yield of PSII (*Φ*_PSII_), a measure of the rate of linear electron transport driving photosynthesis and photorespiration when leaves are illuminated and photosynthesis is activated, is shown in Figure 4C. No substantial differences between P_10_- and SWCNT_10_-treated leaves were observed; however, *Φ*_PSII_ was lower in SWCNT_100_ and SWCNT_300_ variants in comparison to P_100_ and P_300_, respectively. *Φ*_PSII_ was significantly reduced only in SWCNT_300_ plants compared to the controls indicating that the number of photochemical reaction centers able to provide electrons to the photosynthetic electron transport chain [21] was impaired in these plants. An important factor in determining the PSII capability to perform light-induced photochemical reactions is the redox state of Q_A_, which is proportional to the fraction of open PSII reaction centers that are capable of photochemistry [20]. The parameter *q*_L_ which estimates the fraction of open PSII centers and is considered an accurate indicator of the redox state of PSII [20] is presented in Figure 4D. In SWCNT_10_, P_10_ and P_300_ leaves, *q*_L_ was similar to the controls, while it was significantly higher in P_100_ and significantly lower in SWCNT_100_ and SWCNT_300_ when compared to H_2_O-treated plants. No considerable differences were found between SWCNT_10_ and P_10_; however, *q*_L_ was significantly lower in SWCNT_100_ and SWCNT_300_ variants when compared to P_100_ and P_300_, respectively. The lower the value of *q*_L_, the smaller the number of open reaction centers [22], reflecting a lower (reduced) use of light by SWCNT_100_ and SWCNT_300_ plants. These results imply the need for activation of energy dissipation mechanisms with photoprotective function at high SWCNT concentrations.

**Figure 5:**
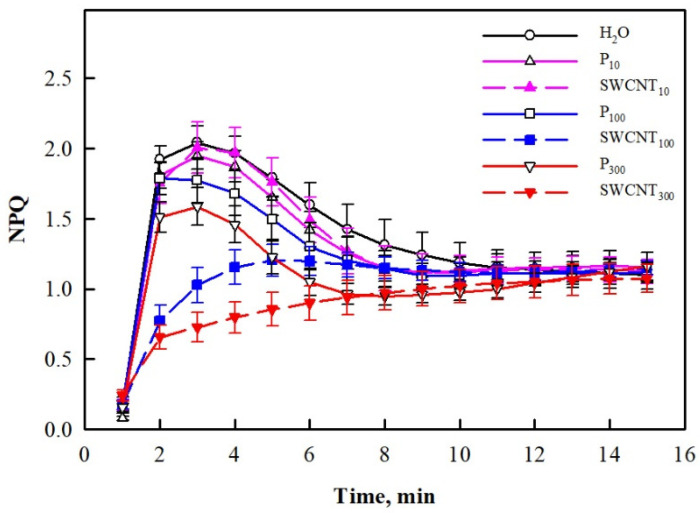
Time course of NPQ development during illumination of pea plants. Data were recorded seven days after spraying with different concentrations of “Pluronic” P-85 (P_10,100,300_) and SWCNTs (SWCNT_10,100,300_). Plants sprayed with H_2_O served as the control. Data are means ± SE (*n* = 6).

We also explored changes in non-photochemical quenching (NPQ) development for a period of 15 min of illumination with actinic (driving photosynthesis) light and established that at the end of this period, NPQ did not differ in any of the treated plants (Figure 5). However, strong differences in NPQ time courses during illumination were established. Sharp increase within the first 3 min followed by a slower decrease until the 10th min and a consequent plateau was observed for control, SWCNT_10_, P_10_, P_100_ and P_300_ variants. It is also to be noted that the beginning of the P_300_ curve had a significantly lower amplitude compared to the controls. In contrast, the peak at the initial NPQ rise was hardly discernible in SWCNT_100_ and completely missing in SWCNT_300_ (Figure 5).

**Figure 6:**
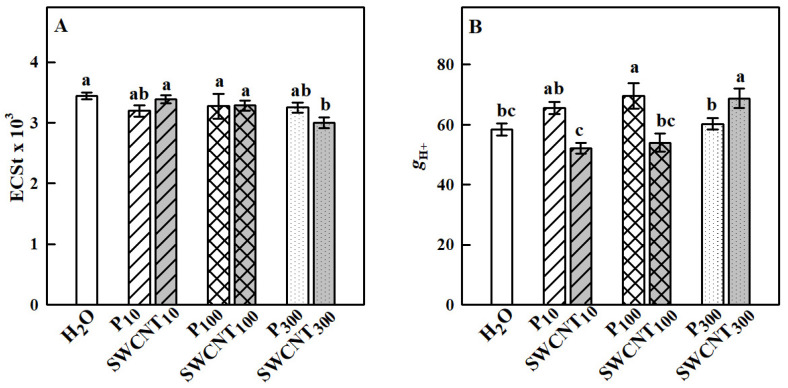
Total size of the thylakoid proton motive force (*pmf*) formed in the light estimated by the ECSt parameter (**A**) and proton efflux (*g*_H+_, **B**) governed mainly by the chloroplast ATP synthase activity. Data were recorded seven days after spraying with “Pluronic” P-85 (P_10,100,300_) and SWCNTs (SWCNT_10,100,300_). Plants sprayed with H_2_O served as the control. Data are means ± SE (*n* = 6). One-way analysis of variance (ANOVA) with a between-subjects factor was performed to assess statistically significant mean differences. Mean separation was performed using a Tukey’s test and statistically different means at the 5% level (*p* < 0.05) are indicated with different letters.

The established differences in the NPQ development process in the studied variants prompted us to explore further two essential components related to its generation—the capability of thylakoid membranes to maintain a large proton motive force (*pmf*) needed for ATP synthesis and operation of the xanthophyll cycle. The electrochromic shift (ECSt) values recorded for SWCNT_10_ and SWCNT_100_ and P_10_, P_100_ and P_300_ leaves were similar to the controls, while they were slightly but significantly lower for the SWCNT_300_ treatment (Figure 6A). In SWCNT_300_ leaves for which ECSt decreased, *g*_H+_ significantly increased compared to H_2_O-treated plants (Figure 6B).

### 2.3. SWCNTs Affect Leaf Pigment Composition

**Figure 7:**
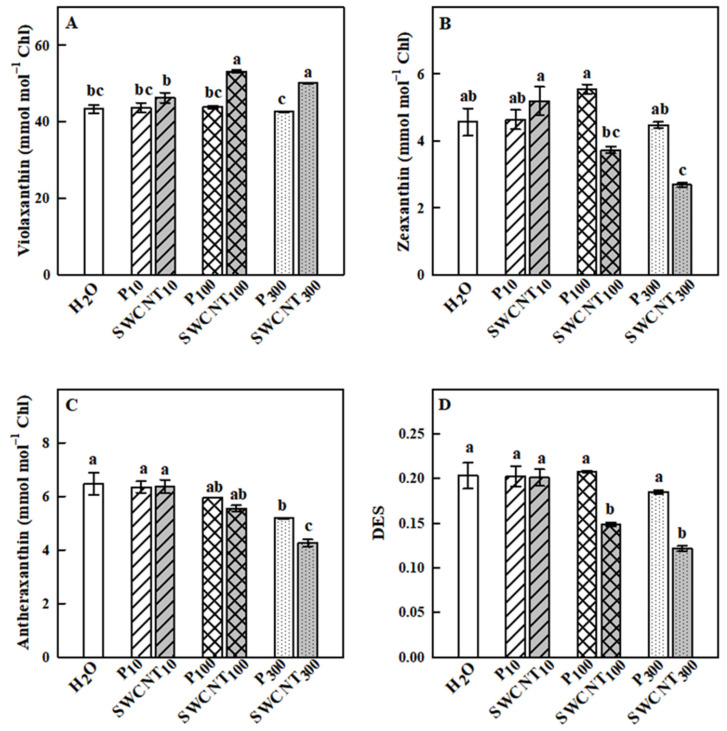
Effect of SWCNTs on xanthophyll cycle pigments. Concentration of violaxanthin (**A**), zeaxanthin (**B**), antheraxanthin (**C**) and the deepoxidation state of xanthophyll pigments (DES, **D**) of pea plants seven days after spraying with different concentrations of “Pluronic” P-85 (P_10,100,300_) and SWCNTs (SWCNT_10,100,300_). Plants sprayed with H_2_O served as the control. Data are means ± SE (*n* = 6). One-way analysis of variance (ANOVA) with a between-subjects factor was performed to assess statistically significant mean differences. Mean separation was performed using a Tukey’s test and statistically different means at the 5% level (*p* < 0.05) are indicated with different letters.

In our study, we performed detailed qualitative and quantitative characterization of the photosynthetic pigments in the plants subjected to P-85 and SWCNT treatments in the light-adapted state. Xanthophyll cycle pigments showed different responses mainly to SWCNT application (Figure 7). Violaxanthin concentration was significantly higher only in SWCNT_100_ and SWCNT_300_ plants in comparison to the controls as well as to P_100_ and P_300_ treatments, respectively, while it remained unchanged in P_10_ and SWCNT_10_ (Figure 7A). Zeaxanthin levels were similar in control, P_10,100,300_ and SWCNT_10_ samples. However, significantly lower zeaxanthin concentrations were detected in SWCNT_100_ and SWCNT_300_ plants (Figure 7B). Antheraxanthin remained unchanged in P_10,100_ and SWCNT_10,100_, but decreased in P_300_ and especially in SWCNT_300_ plants (Figure 7C). SWCNT_100_ and SWCNT_300_ samples exhibited a lower extent of deepoxidation of xanthophyll cycle pigments as judged by the significantly lower deepoxidation index (DES) values than in the other samples (Figure 7D). In the rest of the samples, DES remained unchanged in comparison to the controls.

**Figure 8:**
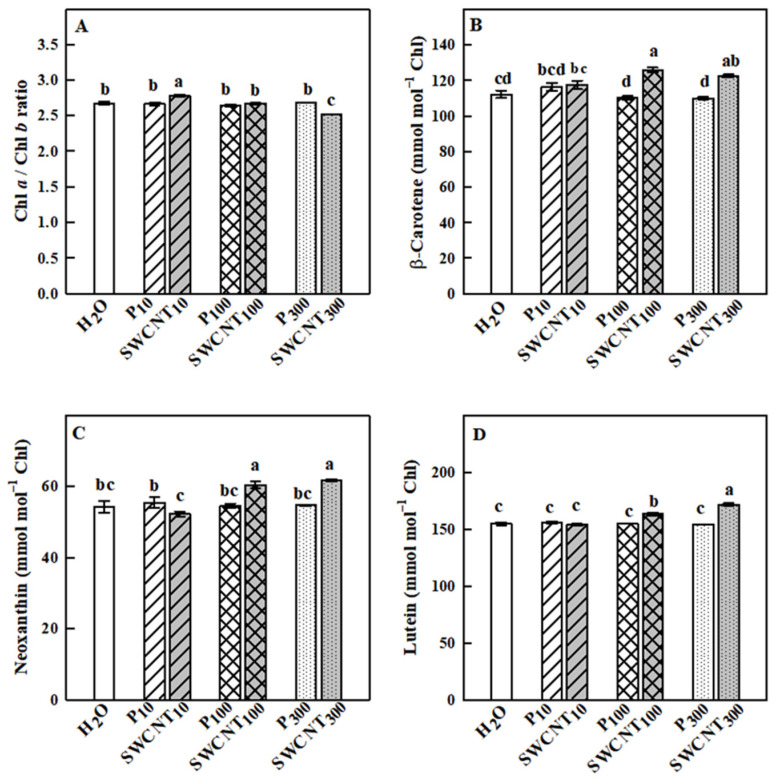
Effect of SWCNTs on chlorophylls and carotenoids. Chl a/Chl b ratio (**A**) and concentration of ß-carotene (**B**), neoxanthin (**C**) and lutein (**D**) of pea leaves seven days after spraying with different concentrations of “Pluronic” P-85 (P_10,100,300_) and SWCNTs (SWCNT_10,100,300_). Plants sprayed with H_2_O served as the control. Data are means ± SE (*n* = 6). One-way analysis of variance (ANOVA) with a between-subjects factor was performed to assess statistically significant mean differences. Mean separation was performed using a Tukey’s test and statistically different means at the 5% level (*p* < 0.05) are indicated with different letters.

The Chl a/Chl b ratio remained relatively stable in response to the applied treatments with some exceptions. Namely, it was slightly but significantly higher in SWCNT_10_ and lower in SWCNT_300_ compared to the controls and the corresponding copolymer treatments (Figure 8A). The changes in the analyzed carotenoids (β-carotene, neoxanthin and lutein), which do not form part of the xanthophyll cycle, are shown in Figure 8B–D. Significant increase in the level of those carotenoids was observed only in SWCNT_100_- and SWCNT_300_-treated plants compared to the controls and the corresponding copolymer treatments. It should be noted that the level of none of the studied pigments changed in the dark-adapted state (data not shown).

## 3. Discussion

The present study revealed multiple concentration-dependent effects of the applied SWCNTs on the leaf structural and functional characteristics, while the copolymer administration did not induce any detrimental alterations, with the sole exception on NPQ dynamics. High doses of SWCNTs affect leaf micromorphology, cause structural alterations in the thylakoid membrane system of chloroplasts, significantly reduce photosynthesis, slow down the generation of the photoprotective state for dissipation of excessive excitation energy and provoke substantial changes in carotenoids. The strongest effects were recorded for SWCNT_300_ samples irrespectively of the method used for characterization and therefore they will be the main discussion point of the next paragraphs.

The foliar application of SWCNT_300_ resulted in accumulation of epicuticular waxes on both adaxial and abaxial leaf surfaces. The biosynthesis of cuticular waxes is a complicated and dynamically regulated process [23]. We speculate that foliar application of SWCNTs could be considered a kind of a mechanical stress effect similar to those provoked by insects. Moreover, cuticular waxes have been recognized as part of the constitutive plant defense against herbivore attacks [24]. The stronger cutinized edge and reduced stomatal opening could limit stomatal conductance, and epicuticular waxes may reduce water loss through the cuticle and/or limit the effect of unfavorable factors such as pathogen infections [25,26]. It is also postulated that leaf epicuticular wax is a physiological drought adaptive mechanism that can improve moisture stress tolerance of pea plants [27]. Long-chain fatty acids, one of the main components of epicuticular waxes, are implicated as signal molecules intervening in defense mechanisms [28]. It is interesting to note that the synthesis of acyl chains that are incorporated in waxes occurs in plastids [29], and therefore changes in plastid operation could be expected in turn to reflect wax composition. Alternatively, nanoparticles of different nature with a diameter of less than 43 nm (much larger than the diameter of utilized by us SWCNTs) can easily penetrate stomatal pores as discussed in [30].

A number of studies reported that carbon-based nanoparticles have a potential for penetration through leaves [30]; however, the exact mechanism of SWCNT foliar uptake and localization is not yet documented. Investigations on SWCNT uptake by isolated plant cells/protoplasts in in vitro cultures demonstrated that they are able to cross cell walls and cell membranes through endocytosis [11,31]. Giraldo et al. [12] showed that SWCNTs passively penetrate membranes of extracted chloroplasts and accumulate on thylakoids and stromata. While determining the route through which SWCNTs penetrate the leaves is beyond the scope of the present work, our results clearly illustrate that SWCNT_300_ treatment induced gross changes in the ultrastructure of the thylakoid membrane system leading to swelling of both stroma lamellae and granal thylakoid regions and smearing of grana margins in a concentration-dependent manner. The most pronounced effects were observed in SWCNT_300_ samples. The increase in the grana diameter and modification of the stacked structure in SWCNT_300_ could impede PSII photoprotective mechanisms in different ways. It was suggested that a larger grana diameter may hamper lateral diffusion processes of proteins [32]. Later, this hypothesis was confirmed by Fristedt et al. [33] who demonstrated that phosphorylation of PSII proteins may provoke macroscopic rearrangements of the entire thylakoid membrane network, which can influence lateral mobility of membrane proteins required for repair and sustained PSII activity. The observed structural modifications in SWCNT_100_ and particularly in SWCNT_300_ are consistent with the functional alterations discussed below.

Leaf functional characteristics were also affected by SWCNT treatments in a concentration-dependent manner. Photosynthesis was significantly reduced by both SWCNT_100_ and SWCNT_300_ treatments being stronger in SWCNT_300_ samples. Our results suggest that photosynthesis inhibition in SWCNT_300_-sprayed leaves was mainly due to metabolic limitations evidenced by the increased level of *C*_i_. These limitations are often caused by a decrease in the Rubisco carboxylation efficiency and/or by a reduced level of ribulose-bisphosphate [34]. Photosynthesis inhibition in SWCNT_100_ leaves could be attributed mainly to diffusional limitations. Although the photosynthesis is significantly altered in SWCNT_100_ and SWCNT_300_ samples, it appears that these plants still maintain a high potential for survival evidenced by the fact that the maximum efficiency of PSII in the dark-adapted state is nearly identical to the controls. The present results reveal that PSII is affected by high SWCNT concentrations in a manner that reduces PSII photochemistry and the number of photochemically active PSII centers (either due to the lower amount or to inactivation of some of the open PSII reaction centers). The much lesser *Φ*_PSII_ reduction in SWCNT_100_ and SWCNT_300_ samples compared to photosynthesis inhibition in these plants might be accounted for enhanced photorespiration. Often, the photosynthetic electron transport is reallocated from photosynthesis to photorespiration under unfavorable conditions [35] and the stimulation of photorespiratory metabolism might be useful to protect photosynthesis [36]. In addition to diffusional and biochemical limitations of photosynthesis, the reason for reduced photosynthetic activity could be the accumulation of epicuticular waxes on SWCNT_300_ leaves, which in turn may reduce light absorption by increasing light reflectance.

The NPQ parameter did not change in its relative yield at the end of the illumination period and also in its dynamic development, with the exception of SWCNT_100_ and especially of SWCNT_300_ samples. The well-expressed maximum at the 3rd flash that was visible in the controls was diminished in SWCNT_100_ and SWCNT_300_-treated leaves. The delay in NPQ formation during the illumination cycle could be attributed to: (1) the impaired formation of ΔpH due to changes in thylakoid lumen acidification [37] that could explain the lower ECSt values in SWCNT_300_ samples or (2) to excessive ion efflux from the thylakoid lumen by the ATP synthase [38,39], which is consistent with enhanced *g*_H+_ in SWCNT_300_ thylakoids. It is evidenced that NPQ comprises three components: energy-dependent quenching, qE [40]; state transition quenching, qT, which can divert light harvesting from PSII-associated light-harvesting complexes to PSI [41]; and photoinhibitory quenching, which is caused by accumulation of photodamaged (inactive) centers of PSII [42]. The fast phase of NPQ (τ_1/2_ = 1–2 min) is assigned to the qE component, which is activated by acidification of the thylakoid lumen [40] and strongly depends on the PsbS protein [43,44] and the conversion of violaxanthin to zeaxanthin [45]. Experiments with different *Arabidopsis* mutants identified an additional zeaxanthin-dependent component of NPQ in this timeframe, which is independent of the other quenching components, but strictly depends on the level of zeaxanthin located in the violaxanthin V1-binding site of LHCII trimers, as well as on its epoxidation rate in the dark [44]. In line with this finding, it can strongly be suggested that the slower initial rise of NPQ in SWCNT_100_ and SWCNT_300_ samples is due to impaired violaxanthin deepoxidation and thus due to zeaxanthin binding specifically to the major PSII light-harvesting complex (LHCII). Recently, [46] has evidenced the direct role of zeaxanthin in in vivo aggregation of LHCII; thus, conformational changes in the LHCII due to SWCNT treatments cannot be ruled out. On the other hand, NPQ dynamics in the timeframe of 3–15 min is most probably affected by the level of zeaxanthin bound to minor PSII complexes [44], as well as by possible state transitions induced by the altered thylakoid membrane ultrastructure.

The slower generation of NPQ and decreased *Φ*_PSII_ could be assigned to decreased *pmf* in SWCNT_300_ samples. The decrease in *pmf* is often attributed to increased proton efflux from the lumen through the membrane-embedded ATP synthase as indicated by the higher *g*_H+_ [38,39]. Our results imply that the increase in *g*_H+_, a parameter considered to represent the ATP synthase activity [47], is caused by the disturbed regulation of ATP synthase rather than by photodamage, which is consistent with unaffected *F*_v_/*F*_m_. Alternatively, it can also be affected by cyclic electron flow [38]. Although the physical factor that impairs the generation of *pmf* in SWCNT_300_-treated plants is not directly identified in the present study, interactions between SWCNTs and thylakoid membrane-embedded photosynthetic complexes cannot be excluded. In fact, a number of in vitro studies confirm the physical exchange of electrons between the photosystems and SWCNTs [15,19,48] that might also occur in vivo. The decrease in *pfm* in SWCNT_300_ could be assigned to decreased *Φ*_PSII_, which can be interpreted as lower proton influx by linear electron flow. The *pmf* is also sensitive to cyclic electron flow and the thylakoid membrane conductivity to proton efflux (*g*_H+_). Therefore, the discrepancy between the significant inhibition of photosynthesis, less reduced *Φ*_PSII_ activity and the weaker effect on *pmf* is most probably associated with a more active cyclic electron transport and impaired permeability of thylakoid membranes to protons.

Pigment analysis revealed that the SWCNT_300_ treatment induces an increased level of violaxanthin and decrease in antheraxanthin and zeaxanthin in light-adapted plants, consequently leading to a lower deepoxidation state. The possible explanation for this finding could be that violaxanthin deepoxidation activity is limited by the reduced ascorbate content in chloroplasts [49]. Lower zeaxanthin level in SWCNT_100_ and SWCNT_300_ may diminish the rigidity of thylakoid membranes and increase peroxidative damage [50]. The reduced level of zeaxanthin could be associated with a higher activity of the enzyme zeaxanthin epoxidase which catalyzes the conversion of zeaxanthin to violaxanthin [51], thus compromising the photoprotective mechanisms [52,53]. Indeed, zeaxanthin epoxidase-deficient mutants are characterized by permanent accumulation of zeaxanthin [54]. The observed structural modifications in SWCNT_100_ and particularly in SWCNT_300_ (swelling of stroma lamellae and granal thylakoids and increase in the grana diameter) could also account for the lower level of zeaxanthin in those plants. Indeed, [55] assumed that zeaxanthin epoxidase is mainly enriched in the stroma-exposed regions of the grana stacks and that diffusion of zeaxanthin is a precondition for the conversion of zeaxanthin to violaxanthin. It is postulated that zeaxanthin diffusion could be more restricted in granal membranes than in stroma lamellae due to high protein density [56]. This was also evidenced by the observation that zeaxanthin epoxidation was less efficient in granal membranes than in stromal membranes and the lower efficiency of zeaxanthin epoxidation is accelerated upon unstacking of intact thylakoid membranes [57]. Besides xanthophyll cycle pigments, we also observed an increase in other photosynthesis-related carotenoids, i.e., neoxanthin, lutein and β-carotene, without a significant change in the Chl content. Generally, carotenoids are regarded to function as reactive oxygen species quenchers [50] and an enhanced level of carotenoids correlates with increased resistance to excess light [58]. It has been shown that β-carotene, and to a much lesser extent zeaxanthin, are oxidized by singlet oxygen [59], and the oxidative products of β-carotene may have a role in retrograde signaling, thus further improving stress resistance [60]. Lutein is the most abundant photosynthetic carotenoid, and its specific property is triplet chlorophyll (^3^Chl*) quenching by binding LHCII and other antenna proteins [61,62]. We speculate that the enhanced level of carotenoids could act as an alarm initiating plant defense mechanisms. In order to verify whether reduced zeaxanthin concentration is compensated by increased carotenoid production, plant behavior under enhanced stress conditions (e.g., high light, low temperature) needs to be studied further.

**Figure 9:**
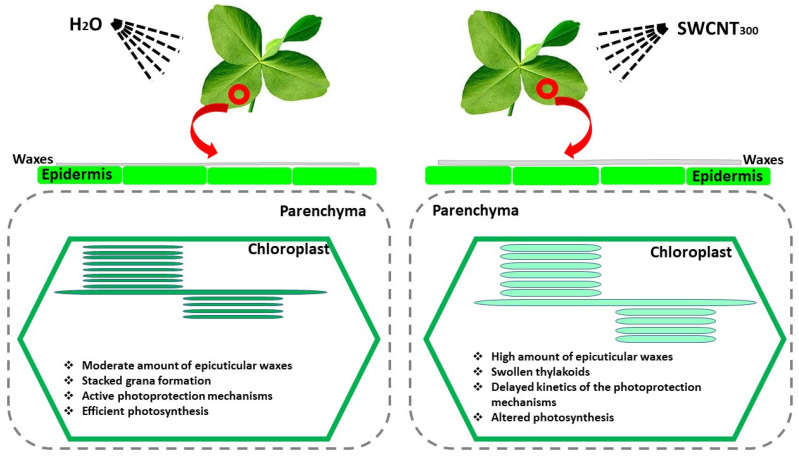
Foliar application of SWCNT_300_ to intact pea leaves provokes accumulation of epicuticular waxes, induces thylakoid swelling and modifies the chloroplast ultrastructure, slows down photoprotection mechanisms and negatively affects photosynthesis. The observed functional, structural and morphological changes are concentration-dependent.

In summary, the present study reveals that foliar application of SWCNTs to intact plants provokes structural changes in leaf micromorphology as well as structural and functional alterations in photosynthetic organelles (chloroplasts). Our data demonstrate that high doses of SWCNTs are associated with the accumulation of epicuticular waxes on both leaf surfaces (only in SWCNT_300_ leaves) and provoke structural modifications of thylakoids in both SWCNT_100_ and SWCNT_300_ samples (Figure 9), which confirms our first hypothesis. These plant responses are accompanied by significant inhibition of photosynthesis, impaired PSII photochemistry, and substantial reduction of the zeaxanthin level in both SWCNT_100_ and SWCNT_300_. The observed swelling of stroma lamellae and granal thylakoids as well as the increase of the grana diameter may impede zeaxanthin diffusion and consequently its conversion to violaxanthin, which is crucial in photoprotection. Our data suggest that SWCNTs might reach and directly affect the photosynthetic machinery, although further investigations are necessary to prove it. We propose that the primary cause for the observed functional changes in SWCNT_300_ samples is the lower *pmf*, as well as the reduced fraction of open PSII reaction centers that are capable of photochemistry [20]. The registered structural and functional alterations positively correlate with SWCNT concentration, supporting our second hypothesis. It is worth noting that the 10 mg L^−1^ SWCNT concentration exhibited very low, if any, effect on the studied photosynthetic characteristics, which makes it an intriguing object for further research related to safe targeted delivery of beneficial substances within the photosynthetic apparatus. The concentration with small negative effects (100 mg L^−1^) might be used as a priming dose, which could stimulate plant defense systems. The application of 300 mg L^−1^ SWCNTs resulted in strong effects that allowed us to detect and analyze in detail the effects that foliar application of those nanomaterials has on the photosynthetic apparatus and leaf morphology.

The findings of the present investigation open up the possibility for detailed studies of the changes in photosynthetic machinery caused by SWCNTs in native plant systems, as well as the use of these carbon nanoparticles as nanocarriers of beneficial substances in the future.

## 4. Materials and Methods

### 4.1. Single-Walled Carbon Nanotubes

Pristine SWCNTs produced by the CVD method (>77% carbon as SWCNTs; diameter × length, 0.7–1.1 nm × 300–2300 nm) were purchased from Sigma-Aldrich (Darmstadt, Germany). The selected method for SWCNT modification is non-destructive and preserves the primary properties of the material [63]. The detailed preparation protocol for SWCNT/polymer composites and the characterization of the mechanical and electrical properties of the nanocomposite materials are detailed elsewhere [63,64]. Briefly, SWCNTs were dispersed in an aqueous medium with the aid of poly(ethylene oxide)_26_-block-poly(propylene oxide)_40_-block-poly(ethylene oxide)_26_ triblock copolymer (“Pluronic” P-85, BASF, Ludwigshafen/Rhein, Germany) (P). The copolymer was dissolved in water prior to adding SWCNTs under sonication. In the aqueous medium, the hydrophobic poly(propylene oxide) blocks adsorb onto the hydrophobic nanotube surface, while the hydrophilic poly(ethylene oxide) chains provide colloidal stability of the system via steric repulsions. SWCNTs (8.2 mg) and a triblock copolymer (820 mg) were added to 28 mL deionized water and gently treated with ultrasound for 15 min affording stable dispersion (SWCNT_300_, 300 mg L^−1^). Then, the concentrated dispersion was diluted with water to obtain samples with SWCNT concentration of 100 (SWCNT_100_) and 10 (SWCNT_10_) mg L^−1^. Three aqueous solutions with corresponding “Pluronic” P-85 concentrations but depleted of SWCNTs (P_300_, P_100_, P_10_) were used in our study for reference. SWCNT dispersions and P-85 solutions were sonicated for 30 min just before the foliar application procedure.

### 4.2. Plant Material

*Pisum sativum* (cv. RAN1) plants were hydroponically grown in a climate chamber under controlled conditions: 150 μmol m^−2^ s^−1^ light intensity provided by SMD LED 6500K light sources, 24/20 °C ± 2 °C day/night temperature, 60–65% relative air humidity and the photoperiod of 12 h. Fourteen-day-old pea plants were sprayed with different concentrations of SWCNTs (SWCNT_10,100,300_) and P-85 (P_10,100,300_). Distilled water was used as for control. For spraying, we always applied 5 mL, which were distributed on the upper leaf surface of well-developed leaf pairs of 20 plants. We were careful to ensure that this amount was evenly distributed and that no drops formed on the leaf surface. Thus, it can be estimated that each leaf pair received no more than 0.4 mL.

After the spraying procedure, the plants were grown for seven more days and all analyses were performed at the end of this period. Ten plants of each treatment were used for non-destructive measurements and the rest of the plants—for destructive measurements, as specified in the text. Dark- and light-adapted pea leaves were detached, frozen in liquid nitrogen and stored at –80 °C until the HPLC analysis of the pigment content.

### 4.3. Scanning and Transmission Electron Microscopy

The micromorphological characteristics (cuticular and epicuticular waxes) of the leaf adaxial and abaxial surface were studied by SEM. The SEM analysis was performed on pre-herbarium leaves in the air-dry state. Leaf lamina was gold-coated with thickness of 0.12 nm using a vacuum evaporator, Jeol JFC-1200 fine coater. Sample observations were performed using a scanning electron microscope Jeol JSM-5510 (Tokyo, Japan) and at least 15 micrographs of each treatment were analyzed.

Chloroplast ultrastructure was studied by transmission electron microscopy following the procedure described elsewhere [65]. Briefly, leaf segments (1 mm^2^) derived from the 2nd and 3rd well-developed leaves were fixed in glutaraldehyde and post-fixed in KMnO_4_. The samples were dehydrated in a gradient of ethyl alcohol (25–100%) and embedded in Durcupan (Fluka, Buchs, Switzerland). Ultra-thin cross-sections were examined by means of an electron microscope (JEOL 1200 EX, Tokyo, Japan). At least 15 micrographs from five plants of each treatment were analyzed.

### 4.4. Photosynthetic Gas Exchange Measurements

Steady-state photosynthesis (*A*_N_), transpiration (*T*), stomatal conductance (*g*_s_) and the ratio of the intercellular–ambient CO_2_ concentration (*C*_i_/*C*_a_) were assessed with a portable LCpro+ photosynthesis system (ADC BioScientific Ltd., Hoddesdon, Herts, UK). Measurements were performed on individual fully expanded leaf pairs (third node from the apical meristem) enclosed into a leaf chamber and exposed to PPFD of 800 μmol m^−2^ s^−1^, flow rate of 200 μmol s^−1^ of air with 400 μmol mol^−1^ of CO_2_ concentration, leaf temperature of 25 °C and 55–60% of relative air humidity.

### 4.5. Chlorophyll Fluorescence Measurements and Analysis of Absorbance Changes

Fluorescence images of intact pea plants were recorded by IMAGING-PAM (MAXI version; Walz, Germany) equipped with an IMAG-MAX/L LED-array illumination unit (blue) and a CCD camera (IMAG-K7, 640 × 480 pixels). The minimum (*F*_0_) and maximum (*F*_m_) fluorescence were determined in 30-min dark-adapted plants and were used to calculate the maximum quantum yield of photosystem II (PSII) as *F*_v_/*F*_m_ = (*F*_m_ − *F*_0_)/*F*_m_. During plant adaptation to the specific light level (180 µmol m^−2^ s^−1^), saturating pulses (over 6000 µmol photons m^−2^ s^−1^ PPFD) with the duration of 0.8 s were applied in order to determine the steady-state fluorescence (*F*′) and the maximum fluorescence (*F*_m_′) in the light. The quantum efficiency of PSII photochemistry (*Φ*_PSII_) was calculated using the formula (*F*_m_′ − *F*′)/*F*_m_′ [21]. The redox state of PSII (*q*_L_), which is a measure of the fraction of open PSII reaction centers was calculated using the formula *q*_L_ = (*F*_q_′/*F*_v_′)/(*F*_0_′/*F*′), where *F*_q_′ is the difference in fluorescence between *F*_m_′ and *F*′ and *F*_v_′ is variable fluorescence from the light-adapted leaf [20]. The minimal fluorescence in the light (*F*_0_′) was estimated using the equation *F*_0_′ = *F*_0_/((*F*_v_/*F*_m_) + *F*_0_/*F*_m_′) [66] and the maximum PSII efficiency in light-adapted leaves was determined as *F*_v_′/*F*_m_′. The non-photochemical quenching parameter (NPQ) reflecting downregulation of PSII as a protective mechanism against excess light was defined according to the equation NPQ = (*F*_m_ − *F*_m_′)/*F*_m_′ [67].

The electrochromic shift (ECS) was determined by MultispeQ V2.0 (PhotosynQ Inc., East Lansing, MI, USA) linked to the PhotosynQ platform (www.photosynq.org) [68]. The amplitude of the first-order decay kinetics of the ECS trace in the first 300 ms (ECSt) was used to assess the proton motive force (*pmf*) [69,70]. The thylakoid conductivity to protons (*g*_H+_) was determined by the ECS dark interval relaxation kinetics at 520 nm [47].

### 4.6. Pigment Analysis

Pigment composition was identified and quantified as described in [71]. The preliminary dark- and light-adapted fresh leaf material was frozen in liquid nitrogen and stored at −80 °C until analyzed. Frozen pea leaves were ground into a fine powder using an MM400 laboratory mill (Retsch, Germany) and resuspended in 2 mL pure acetone. Pigments were extracted for 30 min with continuous shaking at 1000 rpm at 20 °C in the dark. The extract was centrifuged at 11,500× *g* for 10 min at 4 °C, and the supernatant was collected and passed through a PTFE 0.2 μm pore size syringe filter. Quantification of carotenoids was performed by HPLC using a Shimadzu Prominence HPLC system (Shimadzu, Kyoto, Japan) consisting of LC-20AD pumps, a DGU-20A degasser, a SIL-20AC automatic sample injector, a CTO-20AC column thermostat and a Nexera X2 SPD-M30A photodiode array detector. Chromatographic separations were carried out on a Phenomenex Synergi 4 µm Hydro-RP 80Å, 250 × 4.6 mm column. Aliquots of the acetonic extract (20 μL) were injected to the column and the pigments were eluted by a linear gradient from 80% solvent A (acetonitrile, water, triethylamine, in a ratio of 9:1:0.01) and 20% solvent B (ethyl acetate) to 70% solvent B in 11 min at a flow rate of 1 mL/min. The column temperature was set to 25 °C. Eluates were monitored in the wavelength range of 260 nm to 750 nm. Pigments were identified according to their retention time and absorption spectrum and quantified using the integrated chromatographic peak area recorded at the wavelength of maximum absorbance for each kind of pigments using the corresponding molar decadic absorption coefficient [72]. The pigment content was expressed on the Chl basis. The deepoxidation index (DES) reflecting the interconversion of the xanthophyll cycle pigments was calculated according to the following formula:

DES = (antheraxanthin + zeahanthin)/(violaxanthin + antheraxanthin + zeaxanthin).

### 4.7. Statistical Analysis

All data are presented as means ± SE from three independent experiments with at least six biological replicates of each treatment. One-way ANOVA was used to determine differences between treatments (i.e., spraying with either H_2_O, the copolymer or SWCNTs in three different concentrations). Means separation was performed using a Tukey’s test. Significantly different means at the 5% level (*p* < 0.05) are indicated with different letters in the figures.

## Data Availability

The data are contained within the article or the Supplementary Materials.

## Supplementary Materials

The following are available online at https://www.mdpi.com/article/10.3390/ijms22094878/s1, Figure S1: Cuticular and epicuticular waxes at the adaxial (A) and abaxial (B) leaf surface of pea plants seven days after spraying with P_300_; Figure S2: Representative electron micrographs of the chloroplast cross-section taken from intact pea leaves seven days after spraying with different concentrations of “Pluronic” P-85 (A, P_10_; B, P_100_; P_300_, C). G—grana, S—stroma lamellae.
