# Supplementary material for: Single-Walled Carbon Nanotubes Modify Leaf Micromorphology, Chloroplast Ultrastructure and Photosynthetic Activity of Pea Plants
Source: Int J Mol Sci. 2021 May 5;22(9):4878. doi: 10.3390/ijms22094878 (PMC8124974; doi:10.3390/ijms22094878)
Supplement: Supplementary file 1 [file ijms-22-04878-s001.zip › ijms-1194643 (1).pdf]

International Journal of *Molecular Sciences*

MDPI

SUPLEMENRARY FIGURES

Single-walled carbon nanotubes modify leaf micromorphology, chloroplast ultrastructure and photosynthetic activity of pea plants

Violeta Velikova [1,] *, Nia Petrova [2], László Kovács [3], Asya Petrova [1], Dimitrina Koleva [4], Tsonko Tsonev [2], Stefka Taneva [2], Petar Petrov [5], Sashka Krumova [2,] *

[1] Institute of Plant Physiology and Genetics, Bulgarian Academy of Sciences, Acad G. Bonchev Str. Bl. 21, 1113 Sofia, Bulgaria; e-mail@ violet@bio21.bas.bg (V.V.), petrova_assya@abv.bg (A.P.)
[2] Institute of Biophysics and Biomedical Engineering, Bulgarian Academy of Sciences, Acad G. Bonchev Str. Bl. 21, 1113 Sofia, Bulgaria; e-mail@ zlatkova.nia@gmail.com (N.P.), tsonev@gmail.com (T.T.), sgtaneva@gmail.com (S.T.), sashka.b.krumova@gmail.com (S.K.)
[3] Institute of Plant Biology, Biological Research Center, Temesvári krt. 62, 6726 Szeged, Hungary; e-mail@ kovacs.laszlo@brc.hu
[4] Faculty of Biology, Sofia University "St. K. Ohridski", 8 Dragan Tsankov, 1164 Sofia, Bulgaria; e-mail@ koleva@biofac.uni-sofia.bg
[5] Institute of Polymers, Bulgarian Academy of Sciences, Acad G. Bonchev Str. Bl. 103, 1113 Sofia, Bulgaria; e-mail@ ppetrov@polymer.bas.bg

* Correspondence: e-mail@ violet@bio21.bas.bg (V.V.), sashka.b.krumova@gmail.com (S.K.)

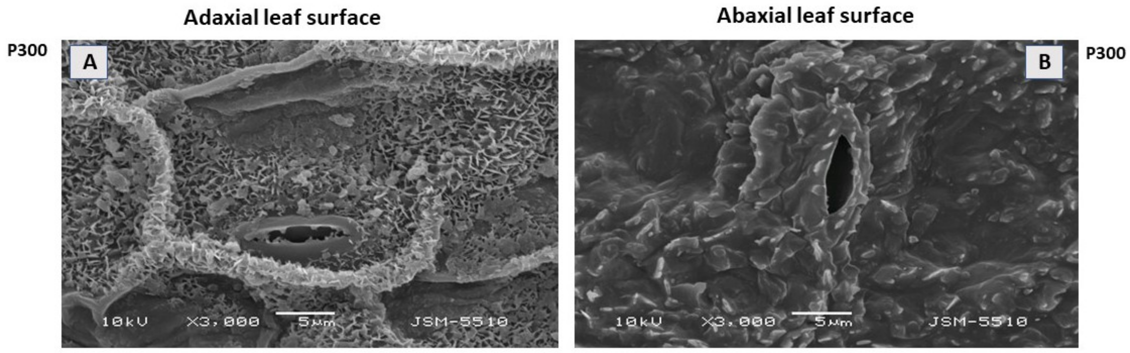

Figure S1. Cuticle and epicuticular waxes at the adaxial (A) and abaxial (B) leaf surface of pea plants seven days after spraying with P_{300}.

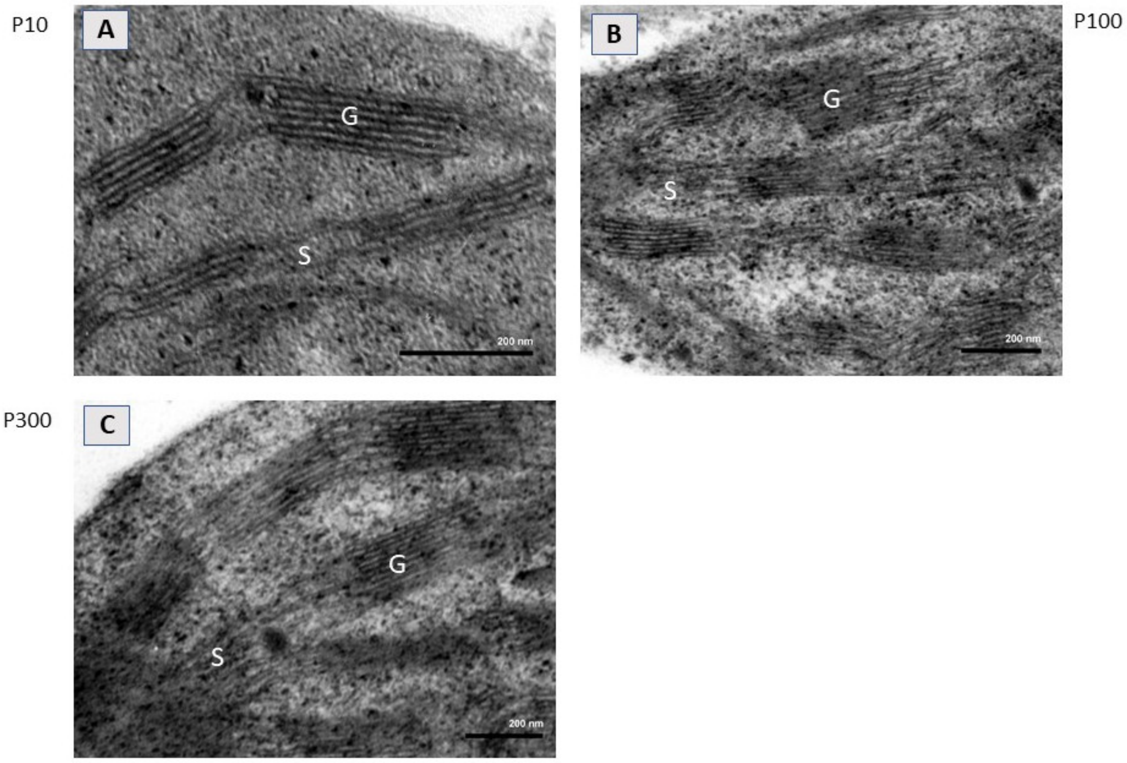

Figure S2. Representative electron micrographs of chloroplast cross section taken from intact pea leaves seven days after spraying with different concentrations of "Pluronic" P-85 (A, P_{10}; B, P_{100}; P_{300}, C). G – grana, S – stroma lamellae.
